# Bone Density Measurement in Anorexia Nervosa: A Missed Opportunity

**DOI:** 10.3390/nu18213617

**Published:** 2026-10-31

**Authors:** Alan Duffy, Dan V. Blalock, Renee D. Rienecke, Thomas Joiner, Philip S. Mehler

**Affiliations:** 1 Eating Recovery Center/Pathlight Mood & Anxiety Centers, 7351 E Lowry Blvd, Denver, CO 80230, USArenee.rienecke@ercpathlight.com (R.D.R.);; 2 Durham Veterans Affairs Medical Center, 508 Fulton St, Durham, NC 27705, USA; 3 Department of Psychiatry and Behavioral Sciences, Duke University School of Medicine, 200 Trent Drive, Durham, NC 27710, USA; 4 Department of Psychiatry and Behavioral Sciences, Northwestern University, 633 Clark Street, Evanston, IL 60208, USA; 5 Department of Psychology, Florida State University, 1879 Miller Landing Rd., Tallahassee, FL 32312, USA; 6 Department of Medicine, University of Colorado School of Medicine, 13001 E 17th Pl, Aurora, CO 80045, USA

**Keywords:** bone density, DXA, clinical inertia, medical complications, refeeding, anorexia nervosa

## Abstract

**Background/Objectives:** Loss of bone mineral density is a common, serious, and often irreversible complication of anorexia nervosa (AN). However, it can be detected by a simple procedure termed dual-energy X-ray absorptiometry (DXA). **Methods:** We review adherence to guidelines, which discuss when this test should be obtained, via a retrospective review of 174 participants treated at a residential treatment center. Electronic health records were reviewed to determine the participants’ most recent DXA. **Results:** Seventy-six people (43.7%) had prior DXAs. Of these, 20 (26.3%) reported their DXAs demonstrated no bone disease. For patients with prior DXA, 48 (63.2%) reported awareness of bone disease, and having any bone disease was significantly more likely than having none (χ^2^ = 11.53, *p* < 0.001). Older patients were more likely to have a prior DXA (OR = 1.07, 95% CI [1.04, 1.10], *p* < 0.001). Ten (13.1%) people under age 25 had bone disease. People with a longer duration of illness were more likely to have a prior DXA (OR = 1.08, 95% CI [1.04, 1.12], *p* < 0.001). **Conclusions:** This study demonstrates poor adherence to obtaining DXA testing in AN. This is likely due to “clinical inertia” and lack of clear guidelines, and needs to be addressed to avert long-term adverse sequelae, especially because successful refeeding and weight restoration may not be sufficient to prevent ongoing loss of bone mineral density.

## 1. Introduction

Health care providers have long relied on clinical practice guidelines, or expert opinion when guidelines are lacking, to provide recommendations for patient care. Although there has been concern about the quality of some guidelines [1], clinicians and their patients benefit when high-quality guidelines and expert opinion are adhered to in clinical practice. Loss of bone mineral density is one of the most common medical complications of anorexia nervosa (AN), and one that is unique in that it is potentially irreversible with serious long-term sequelae if not identified and treated early in the course of this illness [2,3]. Thus, some eating disorder current guidelines, based on consensus or expert opinion, recommend that initial bone mineral density testing may be warranted in people with AN who have had amenorrhea for longer than 6–9 months [4,5]. People with AN have frequent contact with different clinicians after their illness is identified, rendering this period of time opportune to order screening densitometry in the form of dual-energy X-ray absorptiometry (DXA), and to judiciously treat those determined to be at high risk for osteopenia or osteoporosis. Therefore, the primary objective of this study was to better understand adherence to bone mineral density testing among care providers involved in treating people with AN, who are at high risk for loss of bone mineral density due to malnutrition and the resultant biochemical abnormalities which are inherent to AN.

## 2. Materials and Methods

This study is a consecutive case series of 174 adult participants treated at an eating disorder treatment center in the United States from June 2023 to July 2024. A retrospective review of the 174 participants’ electronic health records was conducted by an internal medicine physician to determine the participants’ most recent bone mineral density status. The admission history and physical assessment, completed by a physician at the time of admission, were reviewed to determine whether the patient had ever received a DXA scan and, if so, the results. The review also assessed whether female patients were amenorrheic, and whether they had previously been prescribed medications to treat osteopenia or osteoporosis. No actual DXA reports were reviewed, only their mention in the history and physical. Demographic information and the duration of illness were also extracted from the medical record for each participant prior to analyses being conducted. Participants provided informed consent for their records to be reviewed as part of a larger treatment outcomes study approved by Salus Institutional Review Board.

Among these records, 14 patients did not have a clear indication of the presence or absence of a prior DXA, and 3 patients had a diagnosis other than AN restricting or binge-purge subtype. These 3 patients accordingly were not included in analyses, leaving the analytic sample at *N* = 174. Sex assigned at birth comprised 93% female (*n* = 162) and 7% male (*n* = 12). Participants were 92% White/Caucasian (*n* = 160), 3% Hispanic or Latino (*n* = 6), 2% Asian (*n* = 3), 2% Multiracial (*n* = 4), and 1% were Black or African American (*n* = 1). All participants completed a structured clinical interview with a licensed master’s level clinician prior to admission and were admitted to either an inpatient or residential level of eating disorder care. The participants all had a confirmed diagnosis of AN restricting subtype or AN, binge-purge subtype.

### Statistical Analysis

Descriptive statistics including means and standard deviations (for continuous variables) and number of observations and percentages (for categorical variables) were calculated. Univariate logistic regressions with a binomial distribution were used to examine associations among all variables and presence (1) or absence (0) of a prior DXA scan. For categorical predictors, because of low or zero cell sizes, Fisher’s Exact Test was used to supplement logistic regression results. For the dichotomous test of bone disease versus no bone disease in those with a known DXA result, chi-square goodness of fit was used. For logistic regressions, odds ratios and 95% confidence intervals (95% CIs) are reported. All analyses were performed in R version 4.3.3. (R Core Team, 2024).

## 3. Results

See Table 1 for demographic and clinical characteristics of the patients, split by presence of a prior DXA. Of the 174 patients, with an average age of 28.2 (*SD* = 11.2), only 76 (43.7%) had prior DXAs. Of these, 20 (26.3%) reported that their DXAs demonstrated no bone disease. Eight patients (10.5%) with a prior DXA reported an unknown result. For the patients with a prior DXA, 48 (63.2%) reported awareness of bone disease, either in the form of osteopenia or osteoporosis. Among the 68 patients with a prior DXA and a known result, having any bone disease (osteopenia or osteoporosis; 70.6%) was significantly more likely than having no bone disease (29.4%; χ^2^ = 11.53, *p* < 0.001). Of individuals who had a DXA, 9.2% were treated with bone medications. Four received denosumab and three a bisphosphonate.

Older patients were more likely to have had a prior DXA (OR = 1.07, 95% CI [1.04, 1.10], *p* < 0.001), though only three patients were over age 60, and one of these did not have a prior DXA. Conversely, 10 patients (13.1%) under age 25 had osteopenia or osteoporosis. Eating disorder diagnosis was unrelated to likelihood of having a prior DXA (OR = 0.81, 95% CI [0.45, 1.49]). While very few patients without a prior DXA were on bone medications (*n* = 1, 1.0%), the presence of bone medications was associated with a higher likelihood of having had a prior DXA (OR = 9.84, 95% CI [1.70, 186.12], *p* = 0.03; Fisher’s Exact *p* = 0.02). Patients with a longer duration of illness were more likely to have had a prior DXA (OR = 1.08, 95% CI [1.04, 1.12], *p* < 0.001). Among the 135 patients with recorded last date of menses, there was no association between amenorrhea lasting longer than 9 months (versus 9 or fewer months) and the likelihood of a prior DXA (Fisher’s Exact Test *p* = 0.60). Since DXA result is perfectly confounded with having a prior DXA, no more specific test could be performed.

## 4. Discussion

Our findings show very poor adherence to the recommendation to obtain DXA testing, in a cohort of high-risk people with AN, prior to their admission to an eating disorder treatment center. While the vast majority of medical complications associated with AN are reversible with successful nutritional rehabilitation and weight restoration, the loss of bone mineral density stands in stark contrast in that it can be associated with a long-term increased risk of fragility fractures, pain, and loss of height, even when a patient ultimately regains their expected body weight and there has been a resumption of menses [6,7]. This can then result in decades of morbidity given the typically young age of onset of AN [8]. Although our results did demonstrate a better rate of DXA completion in older people with AN and in those with longer durations of illness, these findings are not reassuring because loss of bone mineral density occurs early in the course of the illness and proceeds to decline rapidly. It therefore would have been optimal to find the opposite results. The seminal meta-analysis from ten years ago on bone mineral density, osteoporosis, and fractures among people with eating disorders, found an increased risk of bone disease in people with AN [9]. In one recent large study of patients with AN and a mean age of 32 years, 70% of those tested via DXA were found to have osteoporosis [10]. While the aforementioned concerning results were obtained from 545 inpatients with extreme forms of AN, data obtained from 214 outpatients with milder forms of AN nevertheless similarly revealed that 52% of women had osteopenia, 34% had full-blown osteoporosis, and 30% reported a previous fracture history, with only 14% having had normal bone mineral density at all skeletal sites [11]. The mean age in this study was even younger at 25 years. Equally concerning, a study of 60 adolescent females with a mean age of only 16 years reported that more than 50% already had abnormal bone mineral density scores on DXA testing [12], notwithstanding their young ages and mild forms of eating disorders, compared with the 70% adversely affected in this study. Moreover, other technological testing, besides DXA, has also demonstrated problematic aberrations in bone microarchitecture and diminished bone strength in adolescents with AN [13], again predicting a substantial lifelong increased future risk of fractures [14], with subsequent pain and disability, even in those with a relatively short duration of illness [15].

The refeeding of people with AN has evolved over the last couple of decades. Back then, the approach to weight restoration was very cautious and slow, starting at 10 kilocalories (KCALs) per kilogram (Kg) of body weight. Based in part on Garber’s [16] seminal paper, this process has become more aggressive, wherein 40–50 KCALs/kg is commonly utilized, even for those people with the most extreme forms of the illness. This more rapid approach to weight restoration results in shorter lengths of stay without an increase in adverse events or refeeding syndrome. The registered dietitian (RD) is the nutrition expert who plays a critical role within the multidisciplinary team refeeding the patient with AN [17]. Typically, they use patient data to recommend a target body weight goal and the recommended initial KCALs. Ideal body weight (IBW) is a useful, albeit imperfect, measure to help guide this process. The Hamwi equation has been a valuable tool to calculate IBW based on the equation for women of: Weight (pounds) = 100 lbs + 5 × (Height − 60 inches) [18].

For people with AN who are moderately to severely ill and being treated in a higher level of care (i.e., residential or partial hospitalization), daily calorie prescriptions often begin in the 1400–1800 KCALs/day range wherein the macro composition of their diets is 50% carbohydrates, 30% protein, and 20% fat. To our knowledge, there has never been a published controlled study to define the most optimal diet plan. For those in 24-h care, the goal is to produce 3–4 pounds of weight gain per week, but for those in partial hospitalization, that goal is reduced to 1–2 pounds/week. This is achieved via daily blind weighing and by increasing the calories by 300–400 Kcals every 3–4 days to achieve the weight gain goal [19]. Progressive oral food is the preferred method of refeeding in AN, but oral nutritional supplements, tube feeding, or even total parenteral nutrition (TPN) may occasionally be required and are helpful when used judiciously and with deliberation [20]. But, in the end, calcium supplementation, normalizing low Vitamin D levels, if present, and adequate KCALs to cause expected weight gain are key.

During refeeding, particularly in the earlier phases of weight restoration, it is critical that blood tests are monitored frequently and that results are reviewed in a timely manner. This is required in order to avoid the potentially fatal occurrence of refeeding syndrome, which refers to the potentially dangerous fluid and electrolyte shifts that often occur when reintroducing nutrition after a period of 1–2 weeks of starvation, regardless of the reason for the starvation [21,22]. While refeeding hypophosphatemia is the most common marker of this, refeeding hypokalemia is also common [10], along with abnormalities that can develop in the liver function tests. Phosphorus levels and electrolytes should be obtained every 1–2 days during the first week or so of refeeding for those with moderate and severe degrees of AN, which can become less frequent as this process moves forward. One of the best markers for an increased risk of refeeding syndrome is the body mass index (BMI); the lower the BMI, the higher the risk [23]. The RD should remain an integral member of the treatment team during the full course of care from residential through outpatient care until the patient’s target weight is reached to help ensure achievement of the dietary plan and ongoing weight maintenance [24].

However, in contrast to almost every other body system where its medical complications from AN are reversible with nutritional rehabilitation and weight restoration, the loss of bone mineral density and its resultant osteopenia and osteoporosis will cause longer-term adverse sequelae and never fully normalize.

It is unclear why bone mineral density damage, versus almost all other body systems adversely affected by AN, seems to persist and does not fully recover even with weight restoration and resumption of menses. Part of the answer may be related to the fact that peak bone mass accrual typically coincides with the time period of adolescence that is also most commonly associated with AN’s onset. Therefore, peak bone mass accrual is never reached, which impairs bone health and confers the lifelong increased risk of fragility fractures, vertebral collapse, and loss of height with a resultant stooped posture if not diagnosed early and treated [25]. There are also a number of endocrine factors operational in AN which further exacerbate the bone mineral density injury, including growth hormone resistance [26], elevated serum cortisol levels [27], estrogen and testosterone deficiency [28], along with leptin deficiency [29] to name but a few. Briefly, AN and its malnutrition cause an increase in osteoclast activity resulting in increased bone resorption along with a decrease in osteoblast activity with resultant decrease in bone formation [30,31].

Yet, if DXA scanning were properly obtained early in the course of AN, and if the results demonstrated significant loss of bone mineral density, this information might help patients to further appreciate the severity of their illnesses and the need for timely weight restoration. It would also inform a plan for treatment by their providers, since medicinal options are available to improve bone density in appropriate individuals [32,33,34,35]. This might then impel ongoing serial DXA scan measurements every 1–2 years to monitor the status of their known bone disease.

Given that osteoporosis-related fractures are devastating for patients with AN, and the risk of fractures is long-known, easily identifiable, and potentially mostly preventable with timely intervention, the question is why obtaining DXA testing is so often overlooked in this high-risk and typically young population, as demonstrated in this study? Moreover, even in the general population, less than 20% of patients receive appropriate evaluation and treatment for bone loss even after a fragility fracture is diagnosed [36]. One might posit that since patients with AN are typically young and premenopausal, they do not require DXAs. This is incorrect since the United States Preventive Services Task Force recommends osteoporosis screening via DXA “in younger women identified to be at increased risk of osteoporosis when low body weight is present” [37]. In fact, a most recent publication demonstrating that fracture prevention strategies are effective even in non-eating-disordered young postmenopausal women further supports the need for better identification of at-risk patients with AN [38].

One other explanation for the low rate of obtaining DXAs is that patients were indeed told by their clinician to obtain a DXA but did not follow through. Or perhaps, since published guidelines are a bit nebulous with their DXA recommendations, directives to their patients were inconsistent. Indeed, the optimal management of low bone mineral density in eating disorders, especially in adolescents, is unresolved. Published practice guidelines indicate a lack of definitive recommendations. In addition, nearly 25 years ago Phillips coined the term “clinical inertia.” He intended it to mean a failure to intensify therapy or to treat a medical problem despite recognition of the problem [39]. Thus, simply stated, it is the awareness of a health care problem, but a failure to act. It is illusory to believe that the mere existence of clinical guidelines will prompt appropriate diagnostic and therapeutic action by clinicians. Rather, and unfortunately, the preference for the status quo is pervasive amongst many common areas of medicine, including diabetes care, cancer screening, hypertension control, and lipid-lowering [40,41,42,43], and may be the case in AN, especially due to equivocal guidelines.

In our study demonstrating an overall lack of attention to DXA screening, and to treating loss of bone density in people with eating disorders prior to their admission to a large treatment center, one might rationalize that there would have been substantially better results in “older people” and in those with longer durations of illness. This would be based on the well-known recommendation for osteoporosis screening in all women 65 years of age or older. However, regarding those in their late teens, twenties, and thirties, perhaps providers were not aware of the recommendation to also obtain DXAs in those who are at increased risk for osteoporosis as determined by a formal risk assessment [44]. Low BMI is a clinical variable that should prompt DXA screening even in “younger” people [45]. However, no robust age differentiator was found in this study. Results were very poor in all age groups, even in those with longer durations of illness. Similarly, there was no gender difference favoring females versus males for prior DXA screening as might have been expected.

The exact causative factors that resulted in clinical inertia towards bone density screening opportunities in those with eating disorders are likely multifactorial. Some potential explanations could include a lack of primary care physicians (PCPs) as part of the outpatient care team. There is no substitute for a high-quality PCP managing complex patients [46]. In addition, most residency training programs spend little to no time teaching about the medical issues of people with eating disorders [47,48]. Important knowledge gaps exist due to a lack of exposure to these patients in residency continuity clinics and a resultant lack of ownership of bone disease as a relevant clinical issue. [49] Insufficient knowledge has also been cited as a barrier to providing requisite counseling and care in many other general medical conditions [50]. Another potential explanation for this lack of attention to the bone disease of restrictive eating disorders may be related to a lack of appreciation that there is also inherent danger from just mild loss of bone density, i.e., osteopenia. In fact, most fractures occur with just osteopenia, and even a 1.0 decrease in the DXA T-score result is associated with a doubling of fracture risk when the DXA results are only in the osteopenic range [38]. Moreover, a more proactive approach towards fracture prevention and osteoporosis screening is novel and likely has not been extended to the typically younger age eating disorder population [51,52]. And, finally, the aforementioned somewhat nebulous guidelines are also likely a contributing factor. Thus, given the confluence of the above factors, there is the real potential for uncertainty in regard to appropriate delivery of care related to bone density issues in AN, which might beget the clinical inertia evident in the aforementioned disappointing results.

With the prevalence of eating disorders continuing to rise and affect a relatively younger population, and given that these individuals tend to have frequent contact with dietitians, therapists, psychiatrists, social workers and PCPs during their illnesses, this avails many opportune times for a member of the team to identify this potential issue, and to then order DXA screening and perhaps treat those at high risk for fractures. The adverse and serious sequelae of loss of bone density in restrictive eating disorders are well established. If present, counseling interventions at a minimum could be associated with significant reductions in future risk of fractures and health care-associated costs [53]. And, if advanced osteoporosis or fractures are present, pharmacologic treatments are available and effective even for young people when directed by an informed bone specialist. It is troubling that forty years after this was first identified as a serious issue with AN, there has not been much progress in regard to treatment of osteopenia and osteoporosis, with the need for endocrine referrals to advise about the optimal approach for its treatment.

Clinical inertia is dangerous for patients and difficult to address. Some potential solutions with regard to the bone disease of eating disorders include expanded medical school residency curricula in eating disorders, increased continuing medical education opportunities, use of mobile educational applications, incorporating prompts into the electronic health record, and perhaps, most importantly, the need for high-quality and consistent clinical practice guidelines [54].

There are some important limitations to the current research that should be considered. The data are patient self-report, and not purely reflective of clinician behavior. In addition, all patients were treated in an inpatient/residential setting, and more severely ill patients were not assessed. Yet, the message of this study remains important for providers who treat people with AN.

## 5. Conclusions

This paper demonstrates very poor attention to the daunting medical problem of loss of bone mineral density in those with restricting eating disorders. It is common and consequential and will be increasingly costly and morbid as this population ages. Therefore, this should be a clarion call to all members of eating disorder treatment teams to increasingly, at a minimum, begin screening for loss of bone density with bone densitometry after 9–12 months of amenorrhea or disease activity. There is also a need to embrace a referral process to correctly interpret the results, and to treat judiciously on an individual basis by informed bone disease specialists based on more definitive practice guidelines. Weight restoration and refeeding are certainly important but may not be adequate to prevent loss of bone mineral density. The time is right to improve the bone-related care of patients with restrictive eating disorders.

## Figures and Tables

**Table 1:** Demographic and Clinical Characteristics.

	No Prior *N* = 98 (56.3%)	Prior DXA *N* = 76 (43.7%)	Overall *N* = 174 (100%)
Age (*M*, *SD*)	25.0 (8.5)	32.4 (12.9)	28.2 (11.2)
Gender (*N*, %)			
Female	85 (86.7%)	71 (93.4%)	156 (89.7%)
Male	7 (7.1%)	4 (5.3%)	11 (6.3%)
Trans Female-to-Male	0 (0.0%)	1 (1.3%)	1 (0.6%)
Trans Male-to-Female	1 (1.0%)	0 (0.0%)	1 (0.6%)
Nonbinary	5 (5.1%)	0 (0.0%)	5 (2.9%)
Duration of Illness, Years (*M*, *SD*)	9.2 (8.5)	15.8 (9.9)	12.1 (9.6)
Diagnosis (*N*, %)			
AN-BP	44 (44.9%)	38 (50.0%)	82 (47.1%)
AN-R	54 (55.1%)	38 (50.0%)	99 (52.9%)
Admission BMI (*M*, *SD*)	17.4 (2.4)	16.8 (2.1)	17.1 (2.3)
DXA Results (*N*, %)			
No DXA	98 (100%)	0 (0%)	98 (56.3%)
Not Reported	0 (0%)	8 (10.5%)	8 (4.6%)
No Bone Disease	0 (0%)	20 (26.3%)	20 (11.5%)
Osteopenia	0 (0%)	25 (32.9%)	25 (14.4%)
Osteoporosis	0 (0%)	23 (30.3%)	23(13.2%)
On Bone Medications (*N*, %)	1 (1.0%)	7 (9.2%)	8 (4.6%)
Amenorrhea Duration, Months (*M*, *SD*)	16.4 (30.4)	31.05 (54.1)	21.89 (41.2)
Not Applicable or Missing ^†^ (*N*, %)	18 (18.4%)	21 (27.6%)	42 (22.6%)
Amenorrhea > 9 Months (*M*, *SD*)	26 (26.5%)	21 (27.6%)	51 (27.4%)
Not Applicable or Missing ^†^ (*N*, %)	18 (18.4%)	21 (27.6%)	42 (22.6%)

^†^ Not applicable due to sex, post-menopausal status, or missing date for time calculation.

## Data Availability

The datasets presented in this article are not readily available because participants did not specifically consent to their data being shared with external research groups. Requests to access the datasets should be directed to the corresponding author.
